# Higher immunoexpression of CK14 from the Wnt-1/β-catenin pathway in the development of odontomas

**DOI:** 10.1590/0103-6440202305452

**Published:** 2023-12-22

**Authors:** Glória Maria de França, Leonardo Magalhães Carlan, Hévila de Figueiredo Pires, Cláudia Nunes de Oliveira, Pedro Paulo de Andrade Santos, Hébel Cavalcanti Galvão

**Affiliations:** 1 Postgraduate program of Dental science, Concentration area in Stomatology and Oral Pathology, Federal University of Rio Grande do Norte, Brazil.

**Keywords:** odontogenesis, odontoma, mixed odontogenic tumor, Wnt signaling pathway

## Abstract

Tooth development depends on a series of reciprocal signaling interactions between the oral epithelium and ectomesenchyme. This study aimed to investigate the role of CK14, a protein involved in Wnt-1/β-catenin signaling, in odontogenesis and the development of odontomas. This cross-sectional, retrospective, immunohistochemical study analyzed 30 compound odontomas, 30 complex odontomas, and 17 tooth germs. Higher immunoexpression of CK14 was observed in odontogenic epithelial cells of tooth germs (*p* < 0.001) and odontogenic epithelial cells of odontomas (*p* < 0.001). There was higher immunoexpression of Wnt-1 and β-catenin proteins in epithelial cells of tooth germs (*p* = 0.002 and *p* < 0.001, respectively), as well as in the ectomesenchyme of odontomas (*p* = 0.003 and *p* < 0.001, respectively). β-Catenin was moderately and significantly correlated with CK14 in the membrane of reduced enamel epithelial cells in odontomas (*p* = 0.007). Higher immunoexpression of CK14 was observed in the odontogenic epithelium during the bud and cap stages and lower immunoexpression in the internal enamel epithelium during the bell stage. In odontomas, lower expression of Wnt-1/β-catenin and higher immunoexpression of CK14 were found in odontogenic epithelial cells, especially adjacent to the mineralized material resembling the tooth formed in these lesions.

## Resumo

O desenvolvimento dentário depende de uma série de interações de sinalização recíproca entre o epitélio oral e o ectomesênquima. O objetivo deste estudo foi investigar o papel da CK14 das vias WNT-1/β-catenina na odontogênese e no desenvolvimento de odontomas. Este estudo transversal, retrospectivo, imuno-histoquímico analisou 30 odontomas compostos, 30 odontomas complexos e 17 germes dentários. A CK14 apresentou maior imunoexpressão em células epiteliais odontogênicas de germes dentários (p<0,001) e em células epiteliais odontogênicas de odontomas (p<0,001). A Wnt-1 e a β-catenina apresentaram maior imunoexpressão de proteínas nas células epiteliais dos germes dentários (p = 0,002 e p<0,001, respectivamente), bem como no ectomesênquima dos odontomas (p = 0,003 e p < 0,001, respectivamente). A β-catenina correlacionou-se moderada e significativamente com a CK14 na membrana de células epiteliais reduzidas do esmalte em odontomas (p = 0,007). Maior imunoexpressão da CK14 foi observada no epitélio odontogênico nos estágios de botão e capuz com menor imunoexpressão no epitélio interno do órgão do esmalte no estágio de sino. Nos odontomas, foi observado menor expressão de Wnt-1/β-catenina e maior imunoexpressão da CK14 presente nas células epiteliais odontogênicas, especialmente, vizinhas ao material mineralizado semelhante ao dente formado nessas lesões.

## Introduction

Odontogenesis starts from the dental lamina and depends on a series of reciprocal signaling interactions between the oral epithelium and ectomesenchyme derived from the neural crest^1^ that involve specific signaling molecules, receptors, and transcription factors^2^. The number, size, and shape of teeth are determined during the stages of tooth initiation and morphogenesis; repetitive signaling throughout morphogenesis is responsible for the formation of anomalies in tooth number, size, and shape^3^.

Benign odontogenic tumors are classified based on their histopathological composition into epithelial, ectomesenchymal, or mixed tumors. Mixed odontogenic tumors are characterized by the proliferation of ectomesenchymal and epithelial components^4^ ^,^ ^5^. According to the WHO(2022) classification^5^, mixed tumors include odontomas, ameloblastic fibroma, primordial odontogenic tumor, and dentinogenic ghost cell tumor^4^ ^,^ ^6^.

Odontomas are mixed tumors composed of soft and hard dental tissue. They are generally reported as hamartomas and correspond to the most common benign odontogenic tumors^7^. According to the updated WHO (2022) classification^8^, ameloblastic fibro-odontoma and fibro-dentinoma are included in the odontoma category, with the presence of hard dental tissue formation being usually the first stage during the maturation process, which is more compatible with the development of odontoma^4^.

The Wingless (Wnt-1)/β-catenin signaling pathway is essential for the early activation of odontogenesis^9^. Additional evidence indicates the involvement of this pathway in the development of some odontogenic tumors. The classical Wnt-1/β-catenin signaling pathway, also called the canonical pathway, is usually activated by Wnt-1 and inactivated by Wnt5a. Activation of this pathway stabilizes β-catenin, which results in the cytoplasmic accumulation and nuclear translocation of this protein. In the nucleus, β-catenin participates in the expression of genes involved in the cell cycle both during embryogenesis and during the development of some benign and malignant tumors^10^.

Cytokeratin 14 (CK14) is an intermediate filament typical of odontogenic epithelium and its replacement with CK19 suggests advanced amelogenesis as a consequence of cell secretory activity^11^ ^,^ ^12^. However, this does not occur in odontomas, i.e., CK19 does not replace CK14 in secretory ameloblasts in advanced stages of amelogenesis; thus, the differentiation of ameloblasts is not completed in odontomas^12^. Interestingly, CK14 was found to be immunoexpressed in the epithelial component of ameloblastic fibro-odontomas^13^ ^,^ ^14^ ^,^ ^15^.

Given the above, the present study aimed to investigate what occurs during odontogenesis when a hamartoma is formed instead of a tooth. Higher immunoexpression of CK14 from the Wnt-1/β-catenin pathway may be one of the events involved in the development of odontomas.

## Methods

This is a cross-sectional and retrospective study that compared the presence of CK14, Wnt-1, and β-catenin proteins between tooth germs and odontomas. The sample consisted of 30 compound odontomas, 30 complex odontomas, and 17 tooth germs. The tumor specimens were obtained from the Pathological Anatomy Service of the Discipline of Oral Pathology, Department of Dentistry, Federal University of Rio Grande do Norte (UFRN). The tooth germs were obtained from four fetuses stored at the Laboratory of Pathology, Center of Health Sciences, UFRN, after the mothers had signed the free informed consent form. These fetuses had less than 20 weeks of intrauterine life and weighed less than 500 g.

The study was approved by the Research Ethics Committee of UFRN by Resolution 466/12 of the National Health Council (Approval number 5.144.737/202).

Morphological examination of the hematoxylin/eosin-stained material was performed under a light microscope (Olympus CX31, Olympus Japan Co., Tokyo, Japan) for histopathological characterization of each odontoma case and analysis of the different stages of odontogenesis in the tooth germs. The slides were examined by two pathologists (G.M.F. and H.F.P.) and any disagreement was resolved by a third pathologist (H.C.G.).

### Immunohistochemistry

Three-µm-thick sections were cut from paraffin-embedded tissue blocks. The tissue sections were deparaffinized and immersed in 3% hydrogen peroxide to block endogenous peroxidase activity. The sections were then washed in phosphate-buffered saline. For the steps of deparaffinization, rehydration, and antigenic retrieval, the sections were incubated with Trilogy (Cell Marque, CA, USA), diluted 1:100 in distilled water, in a Pascal pressure cooker. Next, endogenous peroxidase was blocked with 10 volumes of hydrogen peroxide (15 minutes at room temperature). After washing under running water, the sections were incubated with nonspecific proteins (ThermoScientific, Runcorn, UK) for 5 minutes at room temperature to block nonspecific binding sites. The sections were washed two times (5 minutes each) in Tween 20 plus 1% Tris-HCl (tris-hydroxymethyl-aminomethane, Sigma Chemical Co., St. Louis, MO, USA), pH 7.4. The sections were then incubated with the primary antibodies using antigen retrieval with Trilogy: anti-Wnt (Wnt-1 clone, E-10 monoclonal specificity, sc-514531 Santa Cruz Biotechnology, USA), diluted 1:200, overnight; anti-β-catenin (C2206 clone, anti-β-catenin polyclonal specificity, Sigma Aldrich, USA), diluted 1:2000, overnight, and anti-CK14 (sc-53253, anti-CK14 monoclonal specificity, Santa Cruz Biotechnology, USA), diluted 1:500, 60’. In the next step, the sections were incubated with the HiDef visualization system (CellMarqueTM, USA) using HRP Link as the first reagent and HRP Enzyme as the second reagent, 30 minutes each, interspersed with Tris washes. The reactions were developed with 3,3-diaminobenzidine (Liquid DAB + Substrate, Dako North America Inc., Carpinteria, CA, USA) in a dark room for 5 minutes, followed by washing in distilled water, counterstaining with Harris hematoxylin (5 minutes), washing under running water (5 minutes), and two washes in distilled water (5 minutes). Finally, the sections were dehydrated, cleared, and mounted with a coverslip on Permount resin slides® (Fisher Scientific Inc., Fair Lawn, NJ, USA) for observation under a light microscope. The positive control consisted of human extract of the spinal cord (anti-WNT-1), human carcinomas of the colon (anti-β-catenin), and total HeLa cell lysate (anti-CK14). The negative controls of replacing the primary antibodies with 1% bovine serum albumin in buffer solution.

### Immunohistochemical analysis

Two previously trained pathologists (G.M.F. and H.F.P.) blindly evaluated all cases. The slides were examined under a light microscope at 100x and 400x magnification (Olympus CX31, Olympus Japan Co., Tokyo, Japan). The image was enlarged 400x according to the specifications of the Infinity Analyze® program (Teledyne Lumenera, Ottawa, Canada). Ten representative and consecutive histological fields of each case were photographed using the same program. In these 10 fields, the odontogenic epithelium and ectomesenchymal components were analyzed according to the criteria of Caetano *et al.* ^17^.

Anti-Wnt-1 immunostaining was analyzed quantitatively in the membrane of odontogenic epithelial cells adapted from Santos *et al.* ^18^ Anti-β-catenin immunostaining was analyzed qualitatively according to Halifu *et al*. ^19^ throughout the odontogenic epithelium and ectomesenchymal components of the specimens. Brown staining in the nuclear and membrane compartments was defined as positive. Nuclear and membrane immunostaining for β-catenin was analyzed quantitatively according to Fujii *et al*. ^16^. Anti-CK14 immunostaining was analyzed quantitatively according to the criteria of Wang *et al*. ^20^. The expression of the proteins studied was analyzed based on the number of immunostained cells using the Image J® software, determining the mean number of positive cells per field in the odontogenic epithelium and ectomesenchyme of each case using the following formula: number of positive cells/10 fields. The number of immunostained cells was counted in the nucleus and cytoplasmic membrane for β-catenin, in the membrane for Wnt-1, and the cytoplasm for CK14. To classify semi-quantitatively the immunostained grade (weak, moderate, or strong) of statistical correlations used the parameters: weak (≤ 25% stained cells), moderate (26 - 75% stained cells), and strong (>75% stained cells) adapted from Santos *et al.* ^18^ and examined by two pathologists.

### Statistical analysis

Data were analyzed using the IBM SPSS Statistics freeware (version 20.0; IBM Corp., Armonk, NY, USA). Descriptive statistics were used for the characterization of the sample. The normality and homoscedasticity of the sample were tested by the Shapiro-Wilk test. Since the data were not normally distributed, the nonparametric Kruskal-Wallis test was adopted to determine differences in the antibodies between tooth germs and odontomas, followed by Dunn’s post hoc test for adjusted individual comparisons. The Spearman correlation test was used to determine the correlation between the expression of antibodies analyzed. A level of significance of 5% was adopted for all statistical tests (*p* < 0.05).

## Results

**Figure 1:**
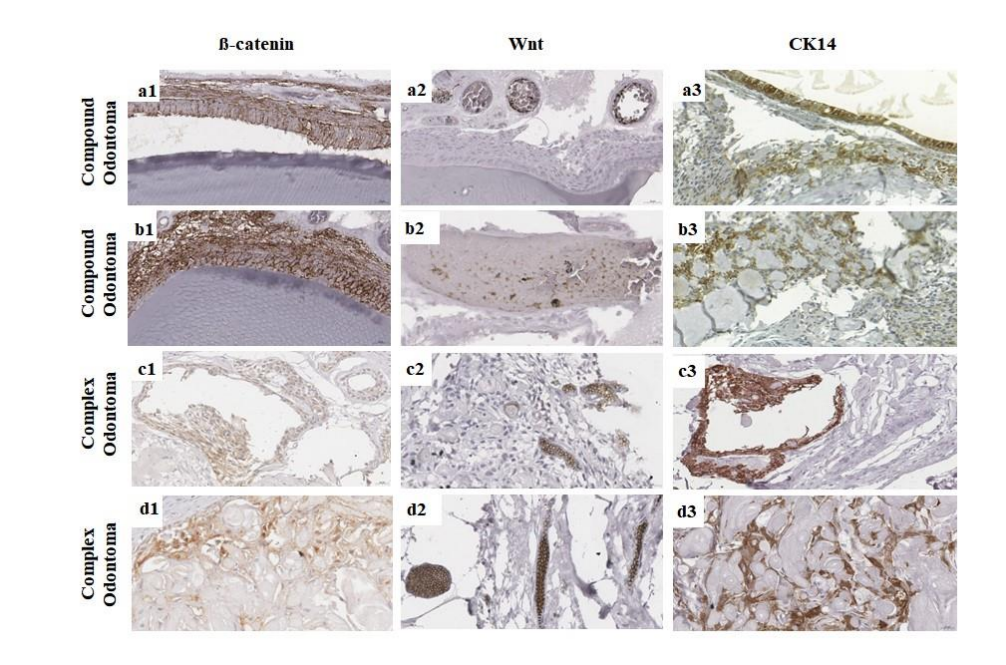
Immunoexpression of β-catenin, Wnt-1, and CK14 in odontomas. Higher immunoexpression of membrane β-catenin in the odontogenic lining epithelium (a1) and ectomesenchyme (b1) of compound odontoma. Higher nuclear immunoexpression of β-catenin in the odontogenic epithelium (c1) and ectomesenchyme (d1) near ghost cells in complex odontoma. Focal immunoexpression of Wnt-1 in islands of odontogenic epithelium in compound odontoma (a2) and complex odontoma (c2) and immunoexpression of Wnt-1 in ectomesenchyme of compound odontoma (b2) and islands of odontogenic epithelium in complex odontoma (d2). Higher immunoexpression of CK14 in the odontogenic folded epithelial lining of compound odontoma (a3) and near to ghost cells of complex odontoma (d3); higher immunoexpression of CK14 in odontogenic epithelial cells of compound odontoma (b3) and complex odontoma (d3) (Scale bar: 20 μm).

Regarding the arrangement of the odontogenic epithelium, histopathological analysis of the cases revealed the presence of reduced enamel epithelium arranged in folded lining (n = 11) and cords (n = 19) and absent odontogenic epithelium (n = 14) in odontomas (Figure 1). Tooth germs were obtained from fetuses at 12, 14, 16, and 17 weeks of intrauterine life. Among the 17 tooth germs located in both the mandible and maxilla, 6 were in the bud stage, 4 in the cap stage, and 7 in the bell stage.

**Figure 2:**
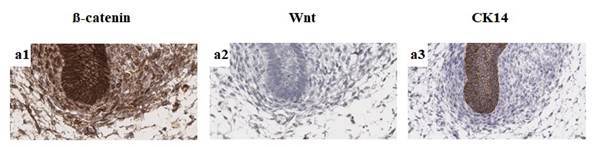
Immunoexpression of β-catenin, Wnt-1, and CK14 in human tooth germs from 12 to 17 weeks of intrauterine life. Bud stage (a1-a3): higher immunoexpression of membrane β-catenin in odontogenic epithelium and ectomesenchyme and higher immunoexpression of nuclear β-catenin in ectomesenchyme. Negative immunoexpression of Wnt-1 and higher immunoexpression of cytoplasmic CK14 in epithelium and negative immunoexpression in ectomesenchyme (Scale bar: 20μm).

**Figure 3:**
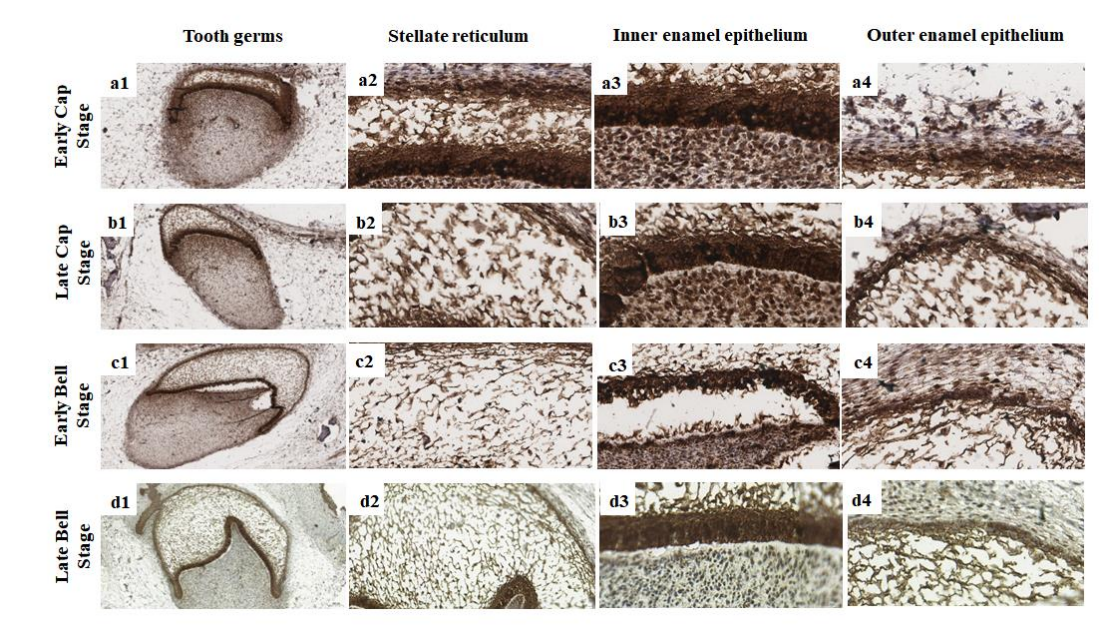
Membrane and nuclear immunoexpression of β-catenin at different tooth germ stages. Higher immunoexpression of β-catenin in odontogenic epithelium and ectomesenchyme during the early and late cap stages (a1, b1), and minor immunoexpression of β-catenin in ectomesenchyme during the bell stages (c1, d1). Higher immunoexpression of nuclear β-catenin in the stellate reticulum at all tooth stages (a2-d2), higher immunoexpression of membrane and nuclear β-catenin in the inner enamel epithelium (a3-d3), and higher immunoexpression of membrane and nuclear β-catenin in the outer enamel epithelium (a4-d4) (Scale bar: 20 µm).

**Figure 4:**
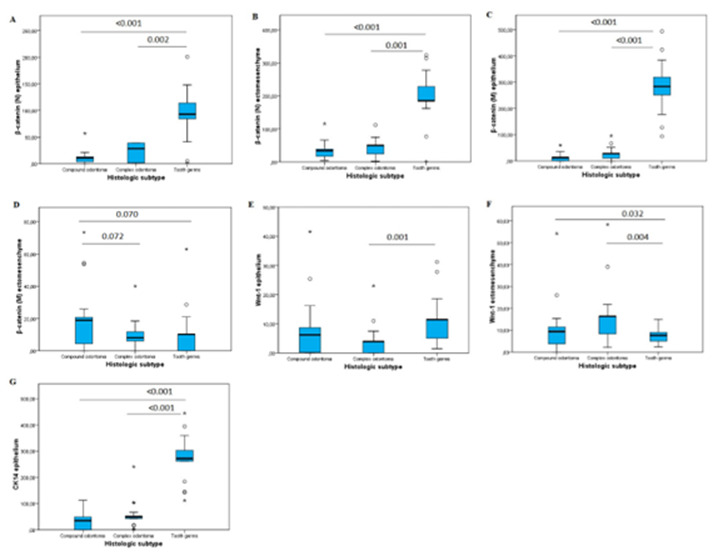
Box plot showing the individual immunoexpression of β-catenin, Wnt-1, and CK14 (Dunn’s post hoc test).

Analysis of the immunoexpression of the antibodies used revealed significant differences for nuclear β-catenin, especially in tooth germs both in the odontogenic epithelium (median = 92.7, *p* < 0.001) and in ectomesenchyme (median = 185.2, *p* < 0.001). Tooth germs showed higher nuclear immunoexpression of β-catenin in odontogenic epithelial cells and ectomesenchyme compared to compound odontoma (*p* < 0.001 and *p* < 0.001, respectively) and complex odontoma (*p* < 0.002 and *p* < 0.001, respectively) (Figure 4). Membrane expression of β-catenin was also higher in the epithelium of tooth germs (median = 283.7, *p* < 0.001). Tooth germs exhibited higher membrane immunoexpression of β-catenin in odontogenic epithelial cells compared to compound odontomas (*p* < 0.001) and complex odontomas (*p* < 0.001) (Figures 1, 2, 3, and 4).

Regarding the compartments where β-catenin was immunoexpressed, intense nuclear immunoexpression was observed in ectomesenchymal cells during the early bud and cap stages and minor nuclear immunoexpression in ectomesenchymal cells at the advanced bell stage; High membrane immunoexpression of β-catenin was observed throughout the odontogenic epithelium at all tooth germ stages, with minor immunoexpression in ectomesenchyme during the bell stage (Figures 2 and 3).

**Figure 5:**
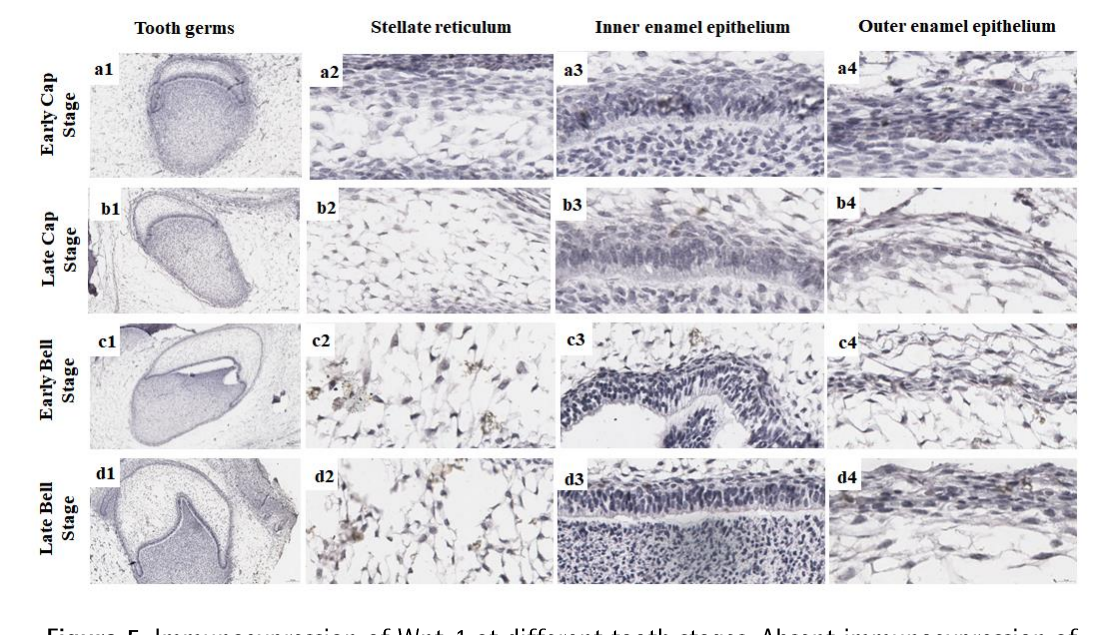
Immunoexpression of Wnt-1 at different tooth stages. Absent immunoexpression of Wnt-1 in stellate reticulum during the cap stages (a2-b2), low and focal immunoexpression of Wnt-1 in stellate reticulum during the bell stages (c2-d2), low and focal immunoexpression of Wnt-1 in the inner enamel epithelium (a3-d3), and focal immunoexpression of Wnt-1 in the outer enamel epithelium (c4-d4) (Scale bar: 20 µm).

Absent immunoexpression of Wnt-1 was seen during the bud stage (Figure 2). There was lower and focal immunoexpression of Wnt-1, especially in the stellate reticulum and external epithelium of the enamel organ, during the bell stages of tooth germs (median = 11.4) (Figure 5) and in ectomesenchyme of complex odontomas (median = 16.3), with statistically significant differences (*p* = 0.002 and *p* = 0.003, respectively) (Figure 1). Tooth germs showed higher immunoexpression of Wnt-1 in odontogenic epithelial cells (*p* = 0.001). There was higher immunoexpression of Wnt-1 in the ectomesenchyme of compound and complex odontomas compared to tooth germs (*p* = 0.032 and *p* = 0.004, respectively) (Figure 4).

**Figure 6:**
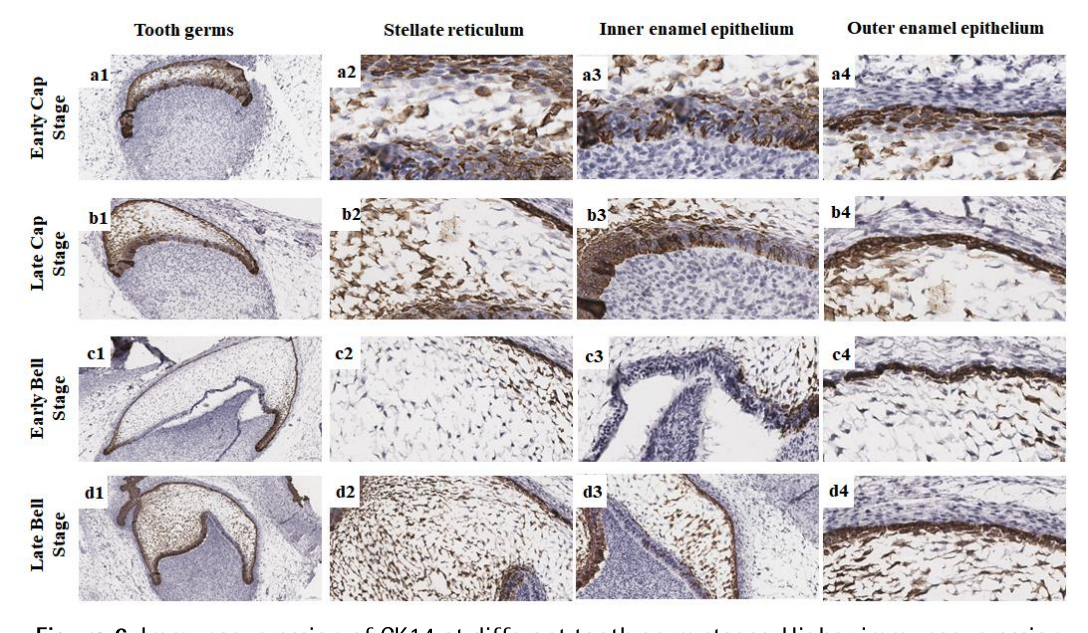
Immunoexpression of CK14 at different tooth germ stages. Higher immunoexpression of CK14 in stellate reticulum at all tooth stages (a2-d2), higher immunoexpression of CK14 throughout the epithelium during the cap stages (a3-b3), minor immunoexpression of CK14 in part of the inner enamel epithelium (c3-d3), and higher immunoexpression of CK14 throughout the outer enamel epithelium (a4-d4) (Scale bar: 20 μm).

Similarly, CK14 was more expressed in odontogenic epithelial cells of tooth germs (median = 271.4, *p* < 0.001), while no immunoexpression was observed in ectomesenchyme (*p* < 0.001) (Figures 2 and 6). Greater CK14 immunoreactivity was found in the odontogenic epithelium of tooth germs (median = 271.4, *p* < 0.001) compared to compound and complex odontomas (Figures 1 and 4G).

Morphologically, different immunoexpression patterns were observed for CK14, particularly the strong immunoexpression of CK14 in odontomas and lower immunoexpression in the inner enamel epithelium of tooth germs during the bell stage, as well as higher nuclear immunoexpression of β-catenin in complex odontomas.

In tooth germs, nuclear β-catenin showed a significant, inversely proportional, and moderate correlation with Wnt-1 (*r* = -0.659, *p* = 0.004) and CK14 (*r* = -0.656, *p* = 0.004) in odontogenic epithelial cells. On the other hand, there was a significant, directly proportional, and moderate correlation of membrane β-catenin with CK14 (*r* = 0.400, *p* = 0.007) in odontogenic epithelial cells of odontomas (Table 1).

**Table 1:** Correlation between immunohistochemical markers analyzed in the odontogenic epithelium and ectomesenchyme of odontomas and tooth germs.

Antibodies	Odontogenic epithelium			
	Odontomas		Tooth germs	
	*r*	*p*	*r*	*p*
Beta-catenin (N) versus Wnt	-0.028	0.856	-0.659	0.004*
Beta-catenin (M) versus Wnt	0.153	0.322	0.424	0.090
Beta-catenin (N) versus CK14	0.117	0.450	-0.656	0.004*
Beta-catenin (M) versus CK14	0.400	0.007*	0.141	0.590
Wnt versus CK14	0.096	0.534	0.223	0.390
Antibodies	Ectomesenchyme			
	Odontomas		Tooth germs	
	*r*	*p*	*r*	*p*
Beta-catenin (N) versus Wnt	-0.012	0.940	-0.377	0.136
Beta-catenin (M) versus Wnt	-0.281	0.064	-0.153	0.557

*Significant correlation (Spearman’s correlation test). Absent immunoexpression of CK14 in ectomesenchyme. N: nucleus; M: membrane.

Additionally, the presence of ghost cells adjacent to the odontogenic epithelium can be observed, especially in complex odontomas with cytoplasmic immunoexpression for CK14 and membrane and nuclear immunostaining for β-catenin (Figure 1: d1 and d3).

## Discussion

The development of a tooth depends on the interactions between the odontogenic epithelium and ectomesenchyme. Four conservative signaling pathways involving SHH, FGF, BMP, and Wnt-1 have been implicated in mediating these tissue interactions during odontogenesis ^21^.

The survival of odontoblasts and the continued production of dentin are ensured in part by endogenous Wnt-1 signaling. The Wnt-1/β-catenin pathway is a key regulator of odontoblast function and the age-related decline in dentin production is therefore due to a decrease in Wnt signaling and the population of Wnt-responsive cells in the pulp ^22^. The present results showed absent Wnt-1 immunoexpression during the early stages of tooth development and focal immunostaining during the bell stages and in odontogenic epithelial cells of odontomas. This fact suggests that the expression of Wnt-1 during odontogenesis and in odontomas contributes to dentin deposition.

During tooth initiation and morphogenesis, Wnt-1 signaling was only detected in the odontogenic epithelium and was absent in ectomesenchyme, while β-catenin signaling was higher during the bud stages. Previous studies indicated that elevated Wnt-1/β-catenin signaling in the epithelium deprived the ectomesenchyme of odontogenic fate *in vivo*, directly suppressing the expression of odontogenic genes and inducing Wnt-1 and BMP antagonists in the odontogenic epithelium ^(^ ^23^. However, when Wnt-1/β-catenin signaling is inactivated in dental ectomesenchyme by deletion of β-catenin, tooth development is arrested at an early stage, suggesting that β-catenin signaling is essential for tooth morphogenesis. The deletion of β-catenin can block tooth morphogenesis by impairing cell mobility ^23^. The lowest expression of Wnt-1/β-catenin was observed in the epithelial component of odontomas that are hamartomas, where less organization or fewer changes in their structure are expected.

In our study, stronger membrane immunoexpression of β-catenin was observed in epithelial cells at all odontogenesis stages, with nuclear β-catenin replacing the membrane form in ectomesenchyme during the bell stages, However, stronger immunoexpression of both membrane and nuclear β-catenin occurred in the epithelial cells and ectomesenchyme of odontomas. Nuclear β-catenin was specifically observed in complex odontomas, indicating higher production of dentin in this type of odontoma. The results suggest that Wnt-1/β-catenin signaling is essential for tooth morphogenesis and cell differentiation and that shifts in the expression of these proteins between odontogenic epithelium/ectomesenchyme can cause developmental disorders, leading to the development of hamartomas.

The Wnt-1/β-catenin pathway and its target, the Lef1 gene, have been associated with the expression of high molecular weight cytokeratins ^11^. CK14 is a protein of the cellular cytoskeleton, which is not directly involved in the APC-Axin-CK1a-GSK3 signaling pathway. However, CK14 can be regulated by different signaling pathways, including the Wnt-1/β-catenin pathway, which is activated by the APC-Axin-CK1a-GSK3 pathway. Within this context, CK14 is considered a downstream target of this signaling pathway since its expression is regulated by this pathway. However, it is important to note that the regulation of CK14 expression is complex and multifactorial, involving several signaling pathways and transcription factors, and cannot be attributed exclusively to the APC-Axin-CK1a-GSK3 pathway ^24^ ^,^ ^25^.

Cytokeratins are divided into two families: one containing relatively large and basic polypeptides (CKs 1-8) and the other containing smaller acid polypeptides (CKs 9-20). CK14 is strongly expressed in the dental lamina, inner enamel epithelium, outer enamel epithelium, stratum intermedium, stellate reticulum, junctional epithelium, reduced enamel epithelium, and epithelial rests of Malassez ^11^. The cervical loop (junction of the outer and inner enamel epithelium) is usually positive for CK14 ^26^.

In general, compound and complex odontomas exhibit immunostaining similar to that of tooth germs, with the odontogenic epithelium expressing high molecular weight pan-cytokeratins (CK14, CK5/6, and AE1/AE3) ^11^ ^,^ ^26^. CK14 is the main cytokeratin of the odontogenic epithelium and intense immunostaining is detected in the odontogenic epithelium of tooth germs and odontoma subtypes, especially ameloblast-like cells ^26^. Accordingly, the present results showed higher immunoexpression of CK14 in the odontogenic epithelium of tooth germs, while in odontomas immunoexpression was higher in odontogenic epithelial cells, similar to two studies that evaluated the expression of CK14 in ameloblastic fibro-odontomas, have reported positivity for this cytokeratin only in the epithelial component of the lesions ^13^ ^,^ ^14^. Additionally, a positive and significant correlation was observed between CK14 and membrane β-catenin expression in odontogenic epithelial cells of odontomas, indicating the role of the Wnt-1/β-catenin pathway that induces the expression of high molecular weight cytokeratins such as CK14 in odontomas ^5^. On the other hand, there was a moderate, significant, and inverse correlation between CK14 and nuclear β-catenin expression in odontogenic epithelium of tooth germs, guiding tooth development.

In odontomas, there was a positive significant correlation between CK14 and membranous expression of β-catenin in epithelial cells. On the other hand, in tooth germs, there was a negative correlation between CK14 and the nuclear expression of β-catenin in epithelial cells. This result suggests that, in tooth germs, β-catenin could negatively regulate the expression of CK14 in epithelial cells. Several signaling functions of β-catenin depend on its cytoplasmic accumulation and subsequent nuclear translocation. Then it can be observed that during dental development β-catenin needs to translocate to the nucleus ^27^, negatively regulating the expression of CK14 in the advanced stages of odontogenesis ^12^. The opposite was observed in odontomas, in which β-catenin remains in the membrane and does not translocate to the nucleus and this allows the persistence of CK14 immunoexpression in these lesions ^11^.

Interestingly, the ghost cells were found within odontogenic epithelium adjacent to immature enamel, as well as, expression of β-catenin was observed in the cytoplasm and nucleus of odontogenic epithelial cells adjacent to the ghost cells in immature odontomas. These findings suggest that odontoma is a hard keratin-expressing tumor-like lesion, especially CK14 and that the Wnt signaling pathway may be involved in the formation of ghost cells in odontomas ^28^. The incidence of ghost cells in complex odontomas is also reportedly higher than that in compound odontomas ^29^ ^,^ ^30^ ^,^ ^31^. In this study, the presence of higher cytoplasmic immunoexpression for CK14 and nuclear immunoexpression for β-catenin was observed in the epithelial cells adjacent to the ghost cells, as reported in the literature.

The highest concentrations of proteins involved in the Wnt-1/β-catenin signaling pathways in the odontogenic epithelium were observed during the early stages of odontogenesis and the shift in expression in the ectomesenchyme occurred mainly during histodifferentiation at the bell stage. However, immunostaining was similar between odontomas and tooth germs. The main difference was the higher immunoexpression of CK14 and the lower expression of β-catenin in odontomas that are hamartomas, where less organization or fewer changes in their structure are expected.
